# Neuroendocrine predictors of vasoplegia after cardiopulmonary bypass

**DOI:** 10.1007/s40618-020-01465-5

**Published:** 2020-11-27

**Authors:** D. Pasero, A. M. Berton, G. Motta, R. Raffaldi, G. Fornaro, A. Costamagna, A. Toscano, C. Filippini, G. Mengozzi, N. Prencipe, M. Zavattaro, F. Settanni, E. Ghigo, L. Brazzi, A. S. Benso

**Affiliations:** 1 Anaesthesia and Critical Care Medicine, Department of Medical, Surgical and Experimental Science, University Hospital, University of Sassari, Sassari, Italy; 2 Division of Endocrinology, Diabetology and Metabolism, Department of Medical Sciences, University Hospital “Città della Salute e della Scienza di Torino”, Turin, Italy; 3 Department of Surgical Science, University of Turin, Turin, Italy; 4 Department of Anesthesiology, Critical Care and Emergency Medicine, Cardiac Intensive Care Unit, University Hospital “Città della Salute e della Scienza di Torino”, Turin, Italy; 5 Clinical Biochemistry Laboratory, University Hospital “Città della Salute e della Scienza di Torino”, Turin, Italy

**Keywords:** Copeptin, Shock, Cardiac surgery, Adrenal insufficiency, NT-proBNP, Vasopressin

## Abstract

**Purpose:**

Vasoplegia often complicates on-pump cardiac surgery. Systemic inflammatory response induced by extracorporeal circulation represents the major determinant, but adrenal insufficiency and postoperative vasopressin deficiency may have a role. Pathophysiological meaning of perioperative changes in endocrine markers of hydro-electrolyte balance has not still fully elucidated. Objectives of the present research study were to estimate the incidence of vasoplegia in a homogeneous cohort of not severe cardiopathic patients, to define the role of presurgical adrenal insufficiency, to evaluate copeptin and NT-proBNP trends in the perioperative.

**Methods:**

We conducted a prospective cohort study in the cardiac intensive care unit of a tertiary referral center. We evaluated 350 consecutive patients scheduled for cardiac surgery; 55 subjects completed the study. Both standard and low-dose corticotropin stimulation tests were performed in the preoperative; copeptin and NT-proBNP were evaluated in the preoperative (T0), on day 1 (T1) and day 7 (T2) after surgery.

**Results:**

Nine subjects (16.3%) developed vasoplegic syndrome with longer bypass and clamping time (*p *< 0.001). Reduced response to low-dose ACTH test was not associated to vasoplegia. Preoperative copeptin > 16.9 pmol/L accurately predicted the syndrome (AUC 0.86, 95% CI 0.73–0.94; OR 1.17, 95% CI 1.04–1.32). An evident correlation was observed at 7 days postoperative between NT-proBNP and copeptin (*r* 0.88, 95% CI 0.8–0.93; *p* < 0.001).

**Conclusion:**

Preoperative impaired response to low-dose ACTH stimulation test is not a risk factor for post-cardiotomic vasoplegia; conversely, higher preoperative copeptin predicts the complication. On-pump cardiac surgery could be an interesting model of rapid heart failure progression.

## Introduction

Postoperative vasodilatory shock is a common complication after major cardiac surgery. It occurs in 5–45% of the procedures and is observed mostly among on-pump intervention [1–4]. This condition has been defined as post-cardiotomy vasoplegic syndrome (PCVS) and is characterized by reduced vascular tone, tissue hypoperfusion and metabolic acidosis [1, 4, 5]. PCVS represents the second cause of vasoplegic shock after sepsis; other well-known associated conditions are major surgical interventions (e.g., organ transplantation), multi-organ failure as a result of burns or multiple traumas, severe pancreatitis [1].

The factors responsible for impaired vasomotor tone after cardiopulmonary bypass (CPB) are only partially understood. An increased incidence of PCVS in patients with a preoperative history of congestive heart failure (HF) has been described and previously attributed to vasodilatory factors such as tumor necrosis factor and nitric oxide [6]. Preoperative use of angiotensin-converting enzyme (ACE) inhibitors has been also independently associated with an increased risk of vasodilatory shock after CPB [2, 6].

Patients undergoing CPB and extracorporeal circulation often experience a systemic inflammatory response syndrome (SIRS) which was associated with both adverse clinical outcomes and alterations in hypothalamic–pituitary–adrenal (HPA) axis function [7, 8]. On this basis, an endocrinological mechanism supposed to be implicated in PCVS is a CPB-related acquired adrenal insufficiency (AI) [8, 9]. Glucocorticoids are necessary for the physiological action of angiotensin II, epinephrine and nor-epinephrine (NE) and, consequently, for maintaining an adequate vascular tone in response to surgical stress [10]. The incidence of post-cardiotomy AI is estimated in 38–60% of surgical procedures, depending on the criteria used for the diagnosis, and is associated to vasopressor resistance with necessity of a prolonged amino-pharmacological support [8]. No evidence about the role of a presurgical HPA axis impairment or exhaustion in the development of PCVS is currently available.

In this setting, vasopressin (AVP) and natriuretic peptides (NPs) represent other major and interdependent regulators of both hydro-electrolyte balance and vascular tone.

The role of circulating AVP levels in the hemodynamics of PCVS patients is complex. In HF, a persistent AVP secretion, mainly due to the reduced tonic inhibition by high-pressure baroreceptors, is involved in the water retention process and in the development of hypotonic hyponatremia [11]. On the other hand, a dysregulation of AVP V1 receptors (V1R), physiologically responsible both for a calcium-mediated vasoconstriction mechanism and for the regulatory feedback on baroreceptor sensitivity, has been hypothesized in critically ill patients and HF [12, 13]. Furthermore, a blunted AVP increase in the early postoperative of patients developing PCVS has been reported, and the cause was attributed to the progressive depletion of the peptide stored in hypothalamic neurogranules under conditions of chronic hyperstimulation [5]. Consequently, in the last years, it has been assumed that a relative AVP insufficiency may contribute to the failure in restoring vascular tone in post-cardiac surgery vasodilatory shock [13–15].

The physiological action of NPs is substantially opposite to that of AVP, promoting natriuresis and vasodilation, also reducing hypothalamic AVP secretion; for its part, AVP is likely to modulate the release of NPs and to regulate their receptors (natriuretic peptides receptor, NPR) activity [16–18]. N-terminal pro-brain natriuretic peptide (NT-proBNP), typically released both in response to acute heart damage (e.g., cardiac ischemia) and myocardial wall stretching, is the most used biochemical parameter among NPs in the clinical management of cardiopathic patients. Both NT-proBNP and copeptin represent stable and reliable markers, respectively, of BNP and AVP secretion; moreover, the combination of these two peptides accurately predicts mortality of patients affected by severe HF [19–21].

The primary aims of the present study were to estimate the incidence of PCVS after CPB surgery and to investigate whether a presurgical impairment of the HPA axis was associated with PCVS. The secondary outcome was to study perioperative copeptin and NT-proBNP changes to explain their pathophysiological role in the onset of vasoplegia.

## Materials and methods

### Study design

We performed a prospective cohort study at the University Hospital “Città della Salute e della Scienza di Torino” in Turin, Italy. The local ethical committee “Comitato Etico Interaziendale” in Turin, approved the study protocol on 12 October 2015 (protocol no. 0099127) and the study has been conducted in accordance with the Declaration of Helsinki.

### Patients

All patients scheduled for CPB cardiac surgery and subsequent admission to the Cardiac Intensive Care Unit were consecutively evaluated for enrollment.

Estimating PCVS incidence equal to 20% of CPB interventions, a population of 50 patients was needed to obtain a 95% CI of 10–33.7%.

Exclusions criteria were: age less than 18 years old, off-pump surgery, cardiac transplantation, extracorporeal membrane oxygenation, dialysis, end-stage hepatic disease, endocarditis, sepsis, septic shock, preoperative use of vasopressors or positive inotropic drugs, chronic or even recent (within the last 2 weeks) corticosteroid therapy, bilateral adrenalectomy and lack of informed consent. All enrolled patients gave their consent to participate to the study.

### Variables and data measurements

In the absence of a widely shared definition, PCVS was diagnosed for the simultaneous occurrence of a mean arterial blood pressure (MAP) < 60 mmHg together with a systemic vascular resistance index (SVRI) < 1200 dyn.s/cm^5^.m^2^, resulting in the need for a NE infusion with dosages ≥ 0.1 µg/kg/min for at least 12 h and within the first 24 h after cardiac surgery [1]. NE and epinephrine, but not dDAVP, were the only vasoconstrictors administered as for clinical protocol in use in our Center.

Before surgery (T0), early in the morning, all patients underwent to HPA axis evaluation with both low-dose (LD) and standard-dose (SD) ACTH test as follows: after baseline cortisol measurement, i.v. Cortrosyn^®^ (cosyntropin, Amphastar Pharmaceuticals, Inc., Rancho Cucamonga, USA) 1 μg bolus was given with collection of venous blood sample for cortisol at 30 and 60 min; immediately after, i.v. Cortrosyn^®^ 250 μg was administrated with cortisol measurement at 120 min. A lack of response was defined for a peak cortisol level < 180 µg/L.

Moreover, at T0, 1 day (T1) and 7 days after surgery (T2), copeptin and NT-proBNP were measured together with routine biochemical analysis; at the same time, hemodynamic monitoring and NE infusion were recorded.

Demographic and clinical data were collected as following: age, gender, EuroSCORE [22], SAPS II [23] and SOFA [24] score, type of surgery, CPB and cross clamping time, preoperative left ventricular ejection fraction (LVEF), anti-hypertensive drugs assumed before surgery (if suspended or not), and renal failure (AKIN classification [25]). In addition, we evaluated if patients needed inotropic and/or vasoactive drugs after CPB and dose.

Finally, the intensive care unit length of stay (ICU-LOS) was reported with follow-up at 28 days.

### Copeptin determination

Blood from an ethylenediamine tetra-acetic acid (EDTA)-containing tube was centrifugated at 4,000 rpm for 5 min and a plasma aliquot was immediately frozen and stored at  − 80 °C until analysis. Copeptin concentrations were determined with the B.R.A.H.A.M.S. KRYPTOR compact PLUS (ThermoFisher Scientific, Hennigsdorf, Germany) automated method. The limit of detection of the assay was 0.9 pmol/L, while intra-assay coefficients of variation were below 7% and below 12% for inter-assay coefficients.

### NT-proBNP determination

Blood samples were collected in an EDTA-containing tube and processed on Cobas e602 automated platform (Roche Diagnostics), including centrifugation at 3500 rpm for 5 min and determination by sandwich immunoassay with two monoclonal antibodies directed against N-terminal portion (1–76) of proBNP molecule (Elecsys proBNP II), using electrochemiluminescence analysis. The limit of detection of the assay was 5 pg/mL (0.6 pmol/L), with a 5–35,000 (0.6–4130) dynamic range as well as intra-assay and inter-assay coefficients of variation of less than 5% at three different concentrations (46, 125 and 14,150).

### Other determinations

All the routine laboratory measurements on serum, plasma and urine samples were performed with automated biochemical assays in the local laboratory (Baldi & Riberi Laboratory, University Hospital “Città della Salute e della Scienza di Torino”, Turin, Italy).

### Statistical analysis

All continuous variables were expressed as mean and standard deviation (SD) or median and interquartile range (IQR), while categorical variables were expressed as number and percentage (%). Inter-group comparisons for continuous variables were performed with the *T* test or the Wilcoxon–Mann–Whitney test depending on type of distribution. The Friedman test was used to identify longitudinal differences. The χ^2^ test or the Fisher’s exact test were used to analyze categorical variables when appropriate; the Pearson coefficient was chosen to evaluate the correlation between continuous ones. The receiver-operating curves (ROC) analysis was used to assess copeptin and NT-proBNP cutoffs able to discriminate patients who developed PCVS. Logistic and linear regression models were calculated to identify possible predictors of PCVS among preoperative or intraoperative variables, as well as to define significant predictors of copeptin and NT-proBNP values over time. ICU-LOS was evaluated as outcome variable. Statistical analysis was performed using Stata/IC^™^ 14.2, version 29 Jan 2018. Figures were made using GraphPad Prism^™^, version 8.01, and MedCalc^™^, version 18.11.3.

## Results

**Fig. 1:**
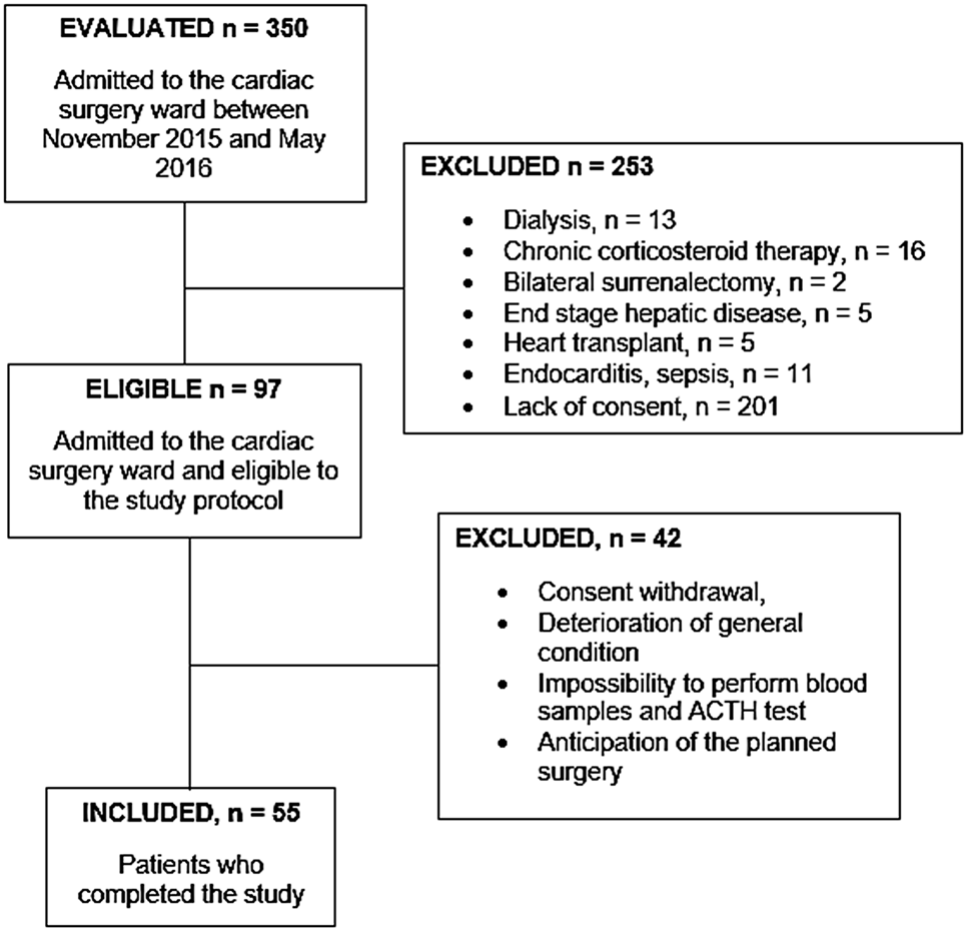
Flow diagram illustrating the selection criteria

We consecutively evaluated 350 patients admitted to the cardiac surgery ward; 253 of them were ineligible according with the exclusion criteria. Among the 97 eligible patients, 55 were enrolled and completed the study (Fig. 1). All relevant demographic variables and the main preoperative data are described in Table 1.

**Table 1:** Demographic and preoperative variables

	All (*n* = 55)	PCVS (*n* = 9)	Not-PCVS (*n* = 46)
Age (years)	72 (60–78)	76 (60–80)	71 (61–78)
Gender (female)	18 (38)	2 (22)	16 (35)
BMI (kg/m^2^)	25.12 (22.8–29)	21.9 (20.7–27.3)	25.3 (23.4–29)
EuroSCORE II	1.28 (0.98–1.83)	1.31 (0.88–1.68)	1.27 (1.03–1.83)
SAPS II	23 (19–28)	31 (19–35)	22 (19–26)
SOFA	4 (2–6)	8 (3–9)	4 (2–6)
LVEF (%)	60 (57–65)	61 (53–65)	60 (57–66)
Severe CKD	5 (9)	3 (33.3)	2 (4.3)^a^
ACEi	21 (38)	5 (55.5)	16 (34.8)
ARB	8 (14.5)	0 (0)	8 (17.4)
Beta-blockers	26 (47)	5 (55.5)	21 (45.6)
Diuretics	27 (49.1)	6 (66.6)	21 (45.6)
Diagnosis			
Mitral valve disease	26 (47)	7 (78)	19 (41)
Aortic valve disease	29 (53)	5 (55.5)	24 (52)
CAD	9 (16)	1 (11)	8 (17)
Surgery			
Mitral valve surgery	24 (43)	7 (78)	17 (37)
Aortic valve surgery	29 (53)	5 (55.5)	24 (52)
Combined valve surgery	7 (13)	3 (33.3)	4 (9)
CABG	12 (22)	3 (33.3)	9 (19)
Cortisol (µg/L)			
ACTH test + 0’	117 (87.1–149.1)	124 (57.2–144.7)	115 (90.3–149.1)
ACTH test + 30’	204 (164–241.3)	164 (113–211.2)	210.1 (165–245)
ACTH test + 60’	181.4 (146–219)	158 (121.3–182.5)	186.2 (147–226)
ACTH test + 120’	262.9 (231–329)	250 (200.7–255.3)	278 (232.9–329.5)

Continuous variables summarized as median and interquartile range (IQR), binary and categorical variables as absolute and percentage values

*ACEi* angiotensin-converting enzymes inhibitor, *ARB* angiotensin receptor blockers, *BMI* body mass index, *CABG* coronary artery bypass graft, *CAD* coronary artery disease, *CKD* chronic kidney disease, *LVEF* left ventricular ejection fraction, *SAPS II* simplified acute physiology score, *SOFA* sequential organ failure assessment

^a^Wilcoxon–Mann–Whitney test, *p* < 0.001

Among the included patients, 9 (16.3%) developed PCVS. Patients who developed PCVS had a significantly longer CPB and clamping time (*p* < 0.001); moreover, they needed more often inotropic support with NE or dobutamine (*p* = 0.008) (Table 2). Mean dosage of dobutamine was slightly higher among patients who developed PCVS compared to those who did not (3.68 ± 2.92 µg/Kg/min vs. 1.42 ± 1.96 µg/Kg/min; *p* = 0.01). The ICU-LOS was longer among PCVS compared to not-PCVS (3 days [2–8] vs. 1 day [1–2]); no patients died during the entire follow-up.

**Table 2:** Intra- and postoperative variables

	All (*n* = 55)	PCVS (*n* = 9)	Not-PCVS (*n* = 46)
CPB (min)	132 (108–158)	170 (149–209)	131 (105–145)^a^
CLAMP (min)	99.5 (82.5–117.5)	137.5 (111–172)	97 (82–107)^a^
Hemodynamic support			
Nor-epinephrine	21 (38)	7 (78)	16 (35)^b^
Epinephrine	4 (7)	2 (22)	2 (4)
Dobutamine	18 (33)	6 (66)	12 (26)^b^

Continuous variables summarized as median and interquartile range (IQR), binary and categorical variables as absolute and percentage values

*CLAMP* clamping time, *CPB* cardiopulmonary bypass

^a^Wilcoxon–Mann–Whitney test, *p* < 0.001

^b^Fisher’s exact test, *p* = 0.008

No pathological response to the SD ACTH test was recorded; moreover, no significant difference was observed neither in basal nor in stimulated cortisol levels between patients who developed PCVS and those who did not (Table 1). Nevertheless, a greater proportion of patients developing PCVS (55.5 vs. 24%) presented a blunted response to the LD ACTH test, although this difference was not statistically significant.

Both copeptin and NT-proBNP significantly increased in the early postoperative respect to basal values. Copeptin at T1 (median 151.8 pmol/L [45.9–311.4]) was higher than at T0 (12.1 pmol/L [7.7–17.6]) and at T2 (12.1 pmol/L [8.7–21.3]; *p* < 0.0001). Differently, NT-proBNP levels increased significantly over the time (T0 median 598 pmol/L [155.5–1255], T1 median 659 pmol/L [276.2–1595.7]), T2 median 1880 pmol/L [956.5–3309.5]); *p* < 0.0001).

**Fig. 2:**
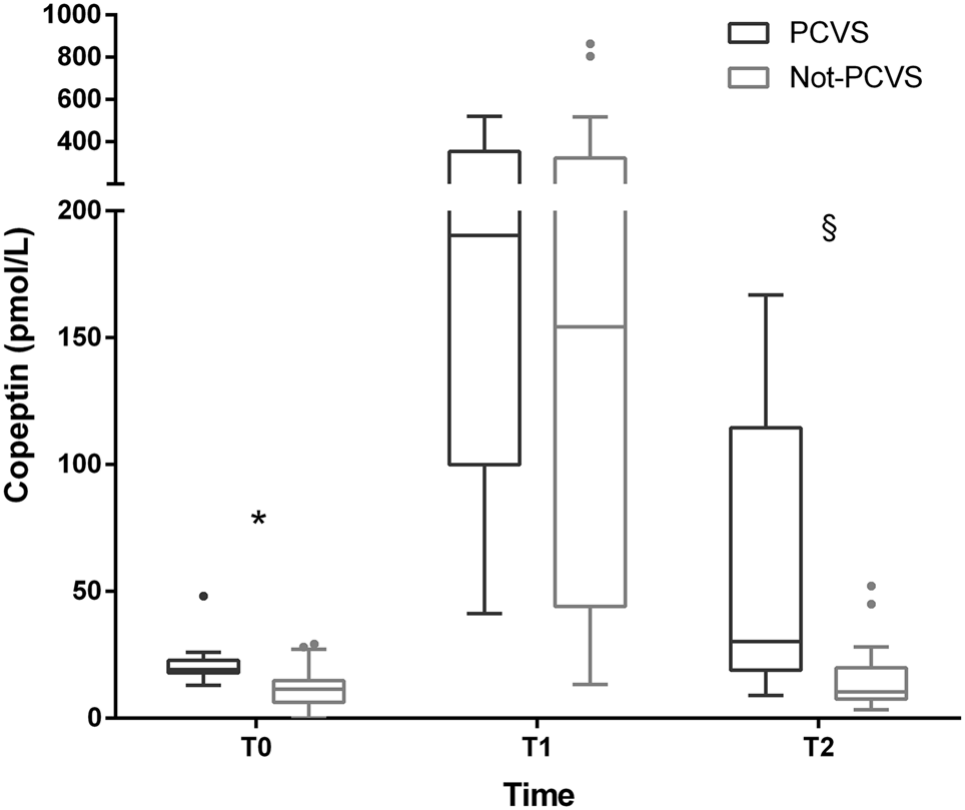
Copeptin at different observation times, patients affected by post-cardiac surgery vasoplegia (PCSV) versus not-PCSV (*p < 0.001; ^§^*p* = 0.002)

Copeptin peak at T1 was similar in PCVS and not-PCVS (Fig. 2). However, copeptin was significantly higher among PCVS both at T0 (19.2 pmol/L [17.89–21.29] vs. 11.39 pmol/L [6.33–14.78]; *p* < 0.001) and at T2 (30.23 pmol/L [19.7–99.85] vs. 10.4 pmol/L [7.63–19.87]; *p* = 0.002) compared to not-PCVS (Fig. 2).

**Fig. 3:**
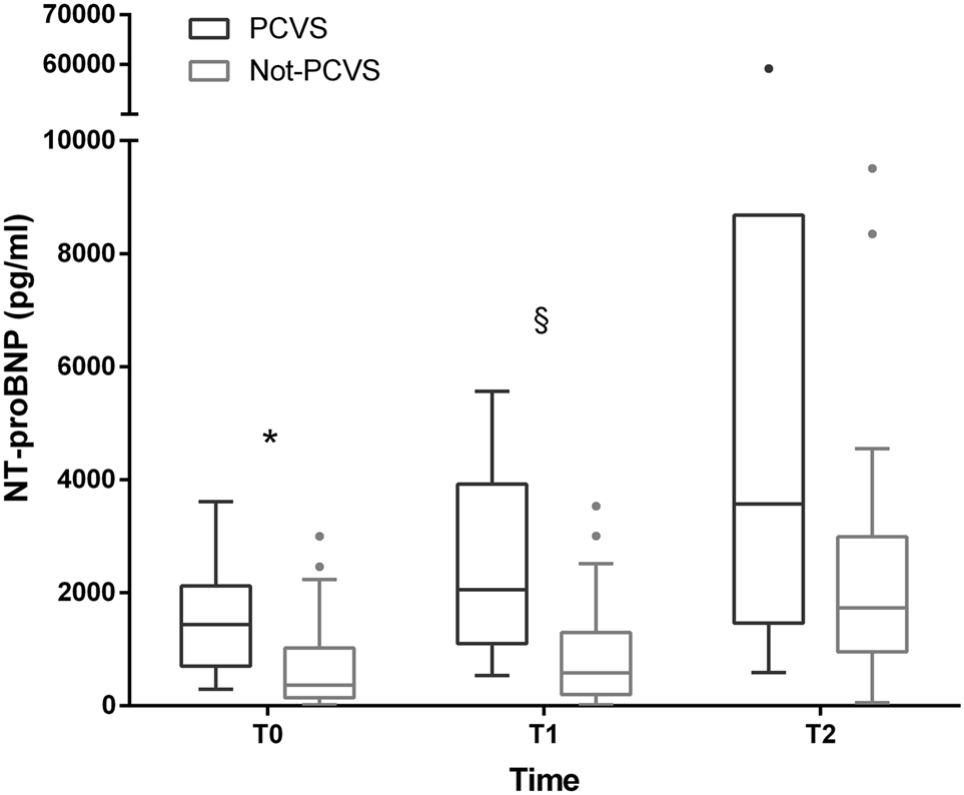
NT-proBNP at different observation times, patients affected by post-cardiac surgery vasoplegia (PCSV) versus not-PCSV (*p = 0.006; ^§^*p* = 0.003)

Finally, NT-proBNP was higher among PCVS at T0 (1435 pg/mL [721.75–1836.25] vs. 365.5 pg/mL [141–977]; *p* = 0.006), at T1 (2,053 pg/mL [1365.25–3465.75] vs. 581 pg/mL [220.5–1,259]; *p* = 0.003), and also at T2, although without a clear significant difference (3571 pg/mL [1687.5–8316] vs. 1733 pg/mL [954–2957]; *p* = 0.06) (Fig. 3).

Despite the main role of AVP and NPs in hydro-electrolyte regulation, no significant difference was observed in serum sodium between the two groups at any time.

At T0, beta-blocker treatment was directly associated with NT-proBNP levels (*r* 0.53; *p* < 0.0001), without interfering with copeptin. At the same time, severe CKD (5 patients, 9.1%) was responsible for an increase in basal copeptin (*r* 0.39; *p* = 0.004) but not in NT-proBNP levels. Finally, diuretic treatment resulted directly associated both to basal NT-proBNP (*r* 0.46; *p* = 0.0004) and copeptin (*r* 0.36; *p* = 0.008).

At T2, beta-blocker treatment was confirmed as a significant predictor (OR 5.2, 95% CI 1.5–18.08) of elevated NT-proBNP levels (> 1880 pg/mL) along with mitral valve surgery (OR 3.88, 95% CI 1.1–13.6). At that time, NT-proBNP showed a marked correlation with copeptin (*r* 0.88 95% CI 0.8–0.93; *p* < 0.001) resulting also the best predictor (OR 1.0005, 95% CI 1.0001–1.001) of higher values of the glycopeptide (> 12 pmol/L), even considering diuretic treatment. Noteworthy, this marked association was not evident either at T0 or at T1.

At univariate logistic analysis, basal copeptin (OR 1.17, 95% CI 1.04–1.32) as well as CPB (OR 1.023, 95% CI 1.005–1.04) and aortic clamp duration (OR 1.043, 95% CI 1.013–1.074) proved to be the best predictors of PCVS. Noteworthy, the association between basal copeptin and PCVS was confirmed at different multivariate models also including CPB (OR 1.15, 95% CI 1.009–1.32), clamping time (OR 1.16, 95% CI 0.996–1.36), severe CKD (OR 1.15, 95% CI 1.021–1.31) or diuretic treatment (OR 1.17, 95% CI 1.03–1.33).

**Fig. 4:**
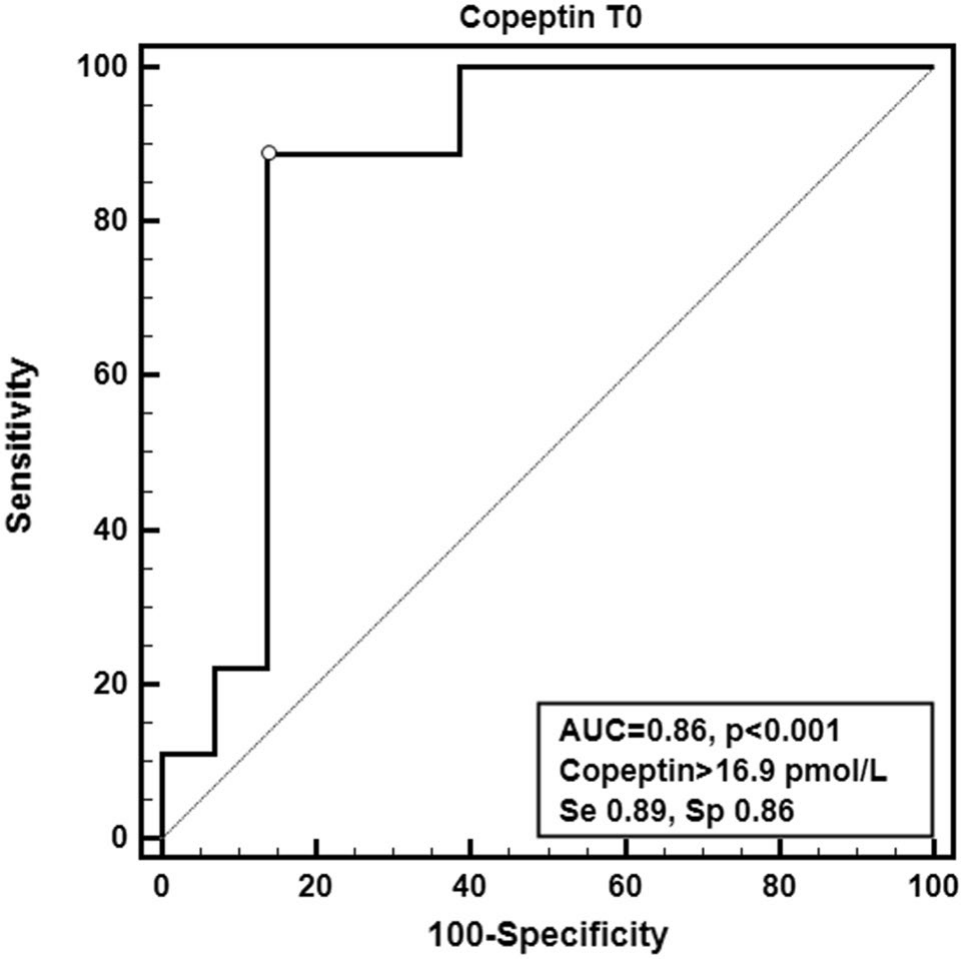
Receiver-operating curve (ROC) analysis calculated for copeptin at T0 associated to the best preoperative cutoff

**Fig. 5:**
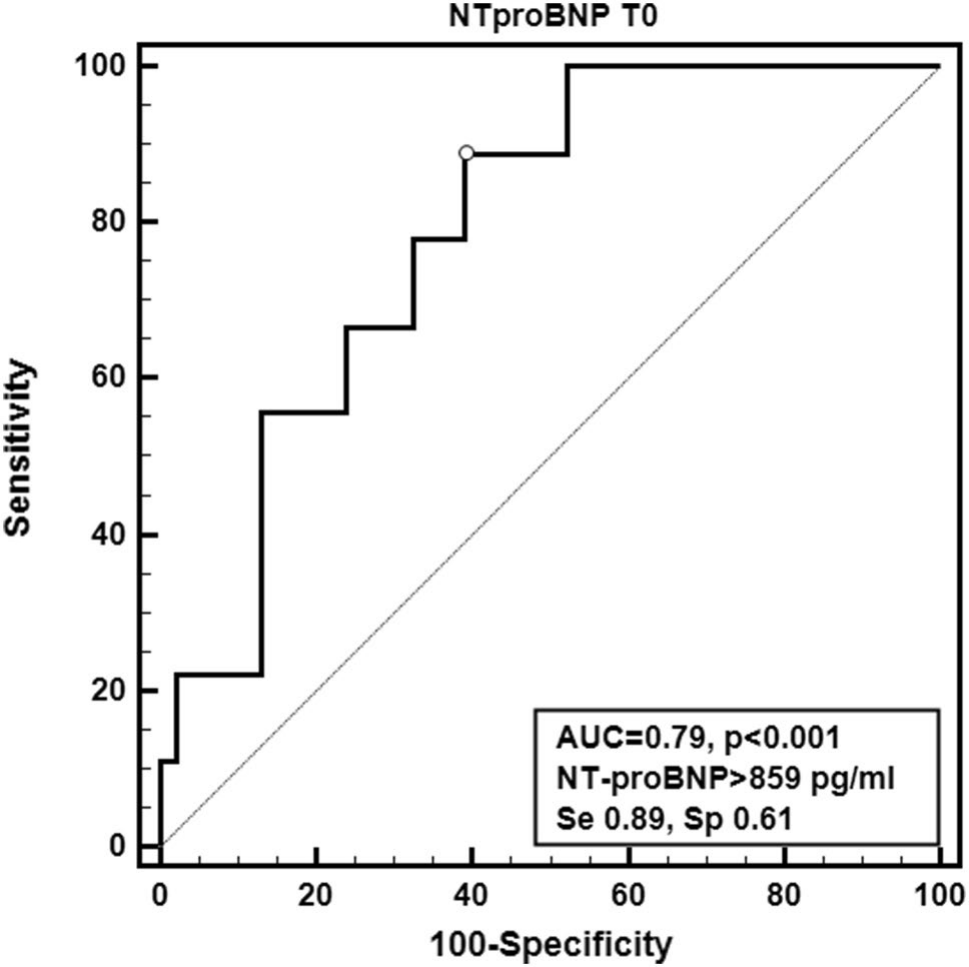
Receiver-operating curve (ROC) analysis calculated for NT-proBNP at T0 associated to the best preoperative cutoff

The ROC analysis identified in a preoperative copeptin value > 16.9 pmol/L cutoff associated to the best accuracy (AUC 0.86, 95% CI 0.73–0.94, Se 0.89, 95% CI 0.52–1.00, Sp 0.86, 95% CI 0.73–0.95) and likelihood ratios (+ LR 6.52, − LR 0.13) in predicting PCVS (Fig. 4). Similarly, the best preoperative cutoff for NT-proBNP was > 598 pg/mL (AUC 0.79, 95% CI 0.66–0.89, Se 0.89, 95% CI 0.52–1.00, Sp 0.61, 95% CI 0.45–0.75, + LR 2.27, − LR 0.18) (Fig. 5). The comparison of the two AUC did not show any significant difference (difference 0.0682, 95% CI − 0.116 to 0.252, *p* = 0.47).

## Discussion

We conducted a prospective study to evaluate possible neuroendocrine predictors of PCVS in a homogeneous cohort of cardiosurgical patients. The observed incidence of PCVS in our population (16.3%) reflects those previously reported in the literature [2–5, 26] and the complication was related to a significantly longer ICU-stay. Furthermore, the subgroup of patients who developed PCVS was comparable to the other in the prevalence of known risk factors such as reduced LVEF, ACE inhibitor treatment or valve surgery (Table 1). Therefore, our population was ideal to investigate the role of possible neuroendocrine determinants of this syndrome.

We found a non-significantly higher percentage of cases of reduced response to preoperative LD ACTH test among patients who developed PCVS. The Society of Critical Care Medicine and the European Society of Intensive Care Medicine have provided an indication in favor of a short-term prophylactic treatment with hydrocortisone in cardiopathic subjects undergoing on-pump surgery [9]. In fact, hydrocortisone, readily available as cortisol, is the only synthetic steroid that has been shown to reduce operative complications such as atrial fibrillation and patient mortality, likely avoiding a Critical Illness Related Corticosteroid Insufficiency (CIRCI) induced by CPB [9]. Unfortunately, our study design did not include a postoperative HPA axis re-evaluation, to define whether these patients developed a CPB-induced CIRCI, although it apparently did not hesitate in an overt PCVS. However, subjects with an history of compromised HPA axis remain to be considered at increased risk for relative corticosteroid insufficiency in case of on-pump surgery.

The other main result of our research is the novel description of two different trends for copeptin and NT-proBNP in the perioperative of such major cardiac interventions. Both are significantly influenced by diuretic therapy in the preoperative period; copeptin is also higher in patients with reduced renal clearance, as expected, while NT-proBNP in those with mitral insufficiency and treated with beta-blockers. Starting immediately after surgery, they take different paths, determined by equally different factors. On the one hand, copeptin is confirmed as an excellent marker of acute stress [27, 28], recording a transient tenfold increase in the early postoperative compared to basal values (Fig. 2). On the other, NT-proBNP immediately reveals the mechanical stress involving the myocardium, remaining elevated even at 7 days (Fig. 3) particularly in those patients who have undergone a procedure involving the valve system.

The further analysis of the PCVS subpopulation still provides new information. First, copeptin confirms its qualities as a prognostic factor in patients suffering from HF, predicting already at baseline the onset of PCVS. This significant association is confirmed by regression analyses also considering interfering therapy with diuretics, severe CKD comorbidity or other well-known determinants of the syndrome such as CPB or clamping time.

Second, our data do not reveal a blunted glycopeptide peak in the immediate postoperative period of PCVS patients, as previously described [5]. Therefore, no evident defect in AVP production is to be suspected in the first postoperative day in case of post-cardiotomic vasoplegia, especially considering the good correlation between AVP and copeptin values previously described during vasodilatory shock [5].

Third, PCVS patients present copeptin levels at 1-week post-surgery almost 50% increased compared to basal ones, unlike non-PCVS. At that time, a strong correlation becomes evident among NT-proBNP and copeptin, even considering diuretic treatment; conversely, these reliable biomarkers of HF progression were not significantly related to each other neither in the preintervention, nor in the early postoperative.

From this point of view, it seems that CPB surgery retraces the neuroendocrine dysregulation observed in severe HF over a truly short period of time. It is not surprising considering that, in few hours, the cardiovascular system of PCVS subjects experiences a prolonged aortic clamp, a complex surgical procedure and a sustained shock induced by endothelial inflammation. Moreover, this novel observation is consistent with the postulated baroreflex dysregulation during intense vascular stress and could be the result of a rapid aggravation of V1R desensitization [13]. In fact, the eventual damage of high-pressure baroreceptors, responsible for the physiological AVP release inhibition, together with the SIRS-related dysregulation in V1R and NPR may be implicated in the observed increase in copeptin and NT-proBNP levels.

This hypothesis implies some other important considerations.

It is possible that long-term monitoring of copeptin in the postoperative of PCVS may be useful to assess the good result of the procedure and patient’s prognosis. In fact, a persistent elevation of this biomarker, able to predict cardiopathic patients’ mortality even in the absence of clinical signs of disease progression [20], could suggest a tighter follow-up because of a possible worse outcome.

Most of all, the increase in copeptin levels at baseline could reveal an advanced stage in the V1R desensitization process as described in severe HF, thus predicting the enormous need for AVP during the PCVS to overcome a rapidly increasing peripheral insensitivity. Recently, several studies have suggested that the use of 1-desamino-8-d-arginine-vasopressin (dDAVP) in this setting would reduce the need for high doses of NE and related adverse effects [29]. A recent meta-analysis showed that the precocious administration of low dose of dDAVP would reduce the rate of perioperative complications in patients undergoing elective and emergency cardiac surgery (OR 0.33, 95% CI 0.20–0.54) [30]. Although a wide heterogeneity was present among the selected papers, the VANCS trial, a double-blind randomized controlled study characterized by the largest sample size, showed a significant reduction in mortality and in postoperative complications in patients treated with dDAVP (0.01–0.06 UI/min) instead of NE (10–60 μg/min) in case of postoperative vasoplegia (unadjusted HR 0.55, 95% CI 0.38–0.80; *p* = 0.0014) [31].

In this context, our data offer a new and more accurate preoperative copeptin cutoff to predict PCVS (> 16.9 pmol/L). In 2011, Colson et al*.* [26] have already suggested that preoperative copeptin  > 9.43 pmol/L could predict CPB-related vasoplegia. Nevertheless, in their cohort, PCVS as well as higher copeptin levels resulted associated to higher cardiovascular functional class, significantly reduced LVEF and hyponatremia; thus, suggesting that post-surgical PCVS is simply predicted by severe HF. Instead, the present copeptin cutoff is not only characterized by an acceptable accuracy and likelihood ratios (AUC 0.86, − LR 0.13), but also is obtained from a homogeneous cohort of cardiopathic patients with preserved LVEF.

Our research has some limitations. First, the protocol was designed to perform the ACTH tests 24 h before surgery, so patients hospitalized on the day of surgery and emergencies were excluded from the study. Second, no HPA axis evaluation was repeated in the first day after the intervention to identify possible CPB-related CIRCI. Finally, the number of PCVS subjects is relatively small, imposing a limited number of covariates at regression analysis.

## Conclusion

Preoperative secondary corticosteroid insufficiency is not an evident risk factor for PCVS and a routinely assessment of HPA axis function might be useless. Nevertheless, hydrocortisone prophylactic treatment in patients undergoing CPB surgery should be considered because of the high risk of CIRCI induced by extracorporeal circulation. Basal preoperative copeptin predicts in our cohort the occurrence of PCVS more accurately than previously reported risk factors. Routine evaluation of preoperative copeptin could be considered to identify high-risk subjects who deserve to be treated with dDAVP in case of vasoplegia.

## Data Availability

The datasets generated and analyzed during the current study are available from the corresponding author on reasonable request.
